# The American Medical Association

**Published:** 1890-06

**Authors:** 


### THE AMERICAN MEDICAL ASSOCIATION.

The American Medical Association's forty-first annual meeting was called to order in the Vendome Theater, Tuesday, May 20, at 11 o'clock.

Dr. W. T. Briggs, chairman of the committee of arrangements, called the meeting to order. The Rev. R. R. Witherspoon offered the opening prayer.

The address of welcome on behalf of the physicians was given by Dr. Briggs.

State Senator Craighead delivered the address in behalf of the State. Mayor McCarver followed on the part of the city.

Dr. Briggs then introduced Dr. E. M. Moore, of Rochester, the President of the association, who delivered his annual address on The Necessity of National Control in Sanitary Matters.

Second Day, Wednesday, May 21.—The general session was called to order at 10 a. m. by President Moore. The Rev. Dr. Winchester delivered the opening prayer.

The nominating committee were then announced.

It was then voted that the word "neurology" be added to the section of Medical Jurisprudence, and also that a vice-chairman as well as chairman be elected to each section.

Dr. N. S. Davis, of Chicago, delivered the address on General Medicine, or the treatment of Fevers by Antipyretics, which was really a plea for the abandonment of the use of alcohol especially, and of the newly introduced synthetic preparations in the treatment of fevers.

The committee on the Rush Monument did not make a very favorable report, but still hoped to be able to lay the cornerstone in 1892, at Washington, D. C.

The trustees of the Journal report an increase in circulation and it now amounts to 4,570.

Third Day, Thursday, May 22.—Meeting called to order by Dr. Moore, followed by prayer from Rev. Dr. Cave.

Committee on nominations reported, recommending: For President, Dr. W. T. Briggs, of Tennessee; First Vice-President, Dr. C. A. Lindley, of Connecticut; Second Vice-President, Dr. R. C. Moore, of Nebraska; Third Vice-President, Dr. H. C. Wyman, of Michigan; Fourth Vice-President, Dr. L. P. Gibson, of Arkansas; Treasurer, Dr. R. J. Dunglison, of Pennsylvania; Secretary, Dr. W. B. Atkinson, of Pennsylvania; Librarian, Dr. C. L. Richardson, of District of Columbia.

Address on Medicine, Dr. E. L. Sherman, of Michigan.

Address on Surgery, Dr. J. M. Mathews, of Kentucky.

Address on State Medicine, Dr. W. L. Schenck, of Kansas.

After a long discussion it was decided to hold the next meeting in Washington on the first Tuesday in May, 1891.

Dr. Samuel Logan, of New Orleans. The doctor stated that the science of medicine had made greater advances in the past half century than in many centuries before, particularly in surgery. The speaker reviewed the relative safety of ether and chloroform in general anæsthesia, giving rules with regard to their administration.

THE SECTION ON PRACTICE, MATERIA MEDICA AND PHYSIOLOGY.

Dr. John H. Musser, of Philadelphia, delivered the annual address. In it he spoke of the greater facilities in treatment enjoyed by the physician, but that it also required more work on the part of the doctor. He also stated that fees were too small in proportion to the work done; that the doctor did himself an injustice by doing so much work, and that he should employ all the labor-saving apparatus possible, such as the telephone, typewriter and phonograph. Every physician should take some rest during each year, as the old "saw" says ,"all work and no play makes Jack a dull boy."

*Filaria*, by Dr. DeSaussure, of Charleston, South Carolina, was quite an interesting paper.

Dr. Lovering, of Ohio, called attention to the similarity of symptoms in trichina and filaria sanguinis hominis.

Dr. C. H. Shephard, of Brooklyn, New York, read an able paper on the Treatment of Rheumatism by means of the Turkish bath, which was discussed by Drs. Ulrich, Didama and Bailey.

Calomel in Dysentery, by Dr. J.W. Davis, of Smyrna, Tenn., was the last paper of the day.

SECOND DAY.

Calomel as a Diuretic, by Dr. George Fackler, of Cincinnati, opened considerable discussion.

Continued Fevers in the South called forth a long but very interesting discussion.

Dr. J. H. VanEnam, of Kansas City, read a paper on the Specific Treatment of Typhoid Fever.

"Has Progress been made in the Medical Treatment of Typhoid Fever?" was the title of an able paper by Dr. T. J. Hoppel, of Trenton, Tennessee.

Dr. H. A. Hare, of Philadelphia, read a paper on the Treatment of Insomnia and Neuralgia by Butyl Chloral Hydrate.

Auto-intoxication in the Presence of an Excess of Nitrogenous Food, by Dr. Sawyer, of Cincinnati, was quite interesting.

THIRD DAY.

Oxygen, its True Value as a Therapeutic Agent, by Dr. J. M. French, was one of the most interesting papers in this section.

Dr. E. L. Schurley's paper on the Local Treatment of Phthisis, was one that is bound to produce good results.

Functional Albuminuria, by Dr. W. B. Davis, was a paper that will prove of great interest to all interested in life insurance.

Dr. Harold M. Mayer, of Chicago, presented an exceptionally able paper on the Clinical Study of Hydrophobia.

Pasteur's Preventive Inoculation of Rabies, was reviewed in a clear and able manner, by Dr. Lagoris, of Chicago.

OBSTETRICAL SECTION—FIRST DAY.

Dr. W. W. Potter, of Buffalo, the President of this section in his annual address gave one of the best in the Convention. He advised that the Secretary be elected for more than one term, he would have an assistant secretary from the place of meeting. He also advised having a stenographer for each section as it was impossible to get the Doctor to write out their remarks even for that most amiable personage, the Journal editor.

Dr. Franklin H. Martin of Chicago, read a very interesting paper, taking for his subject, "A Plea for Early Vaginal Hysterectomy for Cancer of the Uterus; his deductions were drawn from one hundred and thirty-four cases.

This paper called forth a great deal of discussion pro and con. from Drs. Dunning, Graff, Reanny, Wathen and McIntyre·

Second day. Election of officers of Section. President' C. A. L. Reed, of Cincinnati; Secretary, H. A. Kelley, of Baltimore.

Dr. Van der Warker, of Syracuse, N. Y., read a paper on stricture of the urethra in women.

Dr. A. F. Currier, of N. Y., read a paper on a New Operation for the Relief of Prolapsus of the Anterior Vaginal Wall. This was discussed by Drs. Van der Warker, Reanny, Johnston, Engleman and Dunlap.

At this time a telegram was read announcing the sudden death of Prof. W. H. Byford of Chicago.

The Surgical Treatment of Non-Pedunculated Abdominal Tumors, by Dr. Henry O. Marcey, of Boston, was the last paper of this section.

Section on Diseases of Children.—Dr. I. N. Love, of St. Louis, Chairman

By vote the section of Dietetics was invited to meet with this section this meeting.

Dr. G. Frank Lydston, of Chicago, read a paper on Operative Treatment of Acute Peritonitis in Children.

Discussion by Drs. Hare, Larabee, Love, Wood and O'Brien

Acute Rheumatism in Children, by Dr. F. S. Carson, was a general resume of the subject.

Milk Sugar in Infant Feeding was presented by Dr. E. F· Bush. Many papers were read by title this section.

Dr. E. O. Wood, of Pittsburg, Chairman of the Committee on Dietetics, read a very interesting paper on Mastication as a Factor in the Development of the White Race in America.

(To be continued.)

